# Magnetic Resonance Imaging in the Diagnosis of Female Adnexal Masses: Comparison With Histopathological Examination

**DOI:** 10.7759/cureus.42392

**Published:** 2023-07-24

**Authors:** Pooja Ladke, Kajal Mitra, Avinash Dhok, Ameen Ansari, Vrushali Dalvi

**Affiliations:** 1 Radiodiagnosis, N. K. P. Salve Institute of Medical Sciences and Research Centre (NKPSIMS) and Lata Mangeshkar Hospital (LMH), Nagpur, IND

**Keywords:** malignant, ultrasonography, histopathology, magnetic resonance imaging, adnexal lesions

## Abstract

Introduction

Adnexal masses present a special diagnostic challenge because it is difficult to differentiate between benign and malignant lesions clinically. The diagnosis of malignancy is vital, and imaging is the most important part of the evaluation of adnexal masses. This study was conducted with the goal of comparing the accuracy of magnetic resonance imaging (MRI) in diagnosing female adnexal masses in comparison with histopathology examination (HPE). A total of 70 female patients with suspected adnexal lesions were selected for the study. After obtaining informed consent from the patients, an MRI was performed with a subsequent histopathological examination of the lesion.

Results

The study revealed that MRI demonstrated 27% non-neoplastic, 47% benign, and 26% malignant lesions. HPE, the gold standard for identifying and classifying pathological masses, positively identified the lesions and classified them as non-neoplastic, surface epithelial-stromal, germ cell, and sex cord-stromal tumors. The present study of 70 cases with adnexal masses showed a strong positive correlation between MRI and HPE findings.

Conclusion

MRI provides the added advantage of visualization of the tumor matrix with differential identification of the fatty and cystic tissue through heterogeneous signals and enhancement indicating aggressiveness and local spread. MRI has greater diagnostic accuracy when compared to ultrasonography (USG), with HPE as the gold standard for discriminating between benign and malignant adnexal masses.

## Introduction

Adnexal masses present a special diagnostic challenge because it is difficult to differentiate between benign and malignant lesions clinically. The diagnosis of malignancy is vital, and imaging is the most important part of the evaluation of adnexal masses [1]. The majority of adnexal masses removed surgically in women of reproductive age are benign cysts or masses. Although 10% of masses are malignant, many have low malignant potential in patients under the age of 30. Thirty-three percent are mature cystic teratomas, while 25% are endometriomas. The remainder are cystadenomas, either serous or mucinous, or functional cysts [2,3].

Approximately 10% of all ovarian cancers are inherited in the form of hereditary breast-ovarian cancer syndrome or hereditary non-polyposis colorectal cancer syndrome (HNPCC or Lynch syndrome). Malignant neoplasms are infrequent in younger women but become more common as they get older [4]. Ultrasound is the first-line imaging technique for identifying most adnexal masses. Magnetic resonance imaging (MRI) is recommended for the characterization of sonographically indeterminate masses, but computed tomography (CT) is recommended for further staging a suspected ovarian cancer [5,6]. Histopathological examination is the most important and gold-standard means to confirm the type of mass [7].

## Materials and methods

This is a hospital-based cross-sectional study performed in the Department of Radiodiagnosis in a Tertiary care hospital in central India from December 2020 to November 2022. The analysis of the parameters was done after the data had been collected completely. At any point in time, the patients enrolled in the study were given the choice to opt out of the study. The study was initiated only after institutional ethics committee permission was obtained. The study was performed in accordance with the ethical principles specified in the Declaration of Helsinki and as per the guidelines of Good Clinical Practice.

Sample population

The study population included female patients visiting the hospital for evaluation of clinically suspected pelvic lesions. Patients were selected from the target population based on the inclusion and exclusion criteria. Written informed consent was obtained from all enrolled cases in the study. Seventy patients who fit the inclusion criteria entered the study. Inclusion criteria include females who were found to have adnexal masses on ultrasound. The study includes outpatient and inpatient patients during the study period.

Inclusion criteria

Female patients referred to the Department of Radiodiagnosis with clinically suspected pelvic lesions and those who were found to have adnexal masses on ultrasound were included in the study.

Exclusion criteria

All patients for whom surgery was not done or was lost to follow-up were excluded.

Data collecting tools

Imaging Techniques

Informed consent was obtained from all patients. MRI was performed on a 16-channel GE 1.5T HDXT, Version 23.0, with a body coil array used for pelvic imaging.

Assessment by MRI

The patient is asked to fast for three to six hours before the MRI pelvic examination. The patient is lying on her back with a pelvic or torso-phased array coil wrapped tightly around her hip. Images were taken parallel to the uterus in axial, coronal, and sagittal directions. The sequences were taken as shown in Table 1.

**Table 1:** MRI pelvis sequences taken for the study. STIR: short Tau inversion recovery sequence.

Sequence	TR	TE
T1 FS	500–600	40–45
T2WI	5000–5200	80–90
STIR	7000	45–50
Post-contrast	700–750	40–45

In all sequences, the slice thickness was 5 mm, the interslice interval was 1 mm, and the FOV was 30-35. The post-contrast sequence taken was T1WI. Sagittal, coronal, and axial images were acquired whenever indicated. Histopathological diagnosis of the adnexal lesions was obtained from post-operative specimens. The histopathological diagnosis was considered the gold standard.

Data management and analysis procedure

All data were collected, kept confidential, entered into an Excel sheet (Microsoft® Corp., Redmond, WA), and utilized for the analysis. All analyses were calculated using EPI Info Software Version 7. Quantitative data was presented with the help of the mean and standard deviation. Descriptive statistics were evaluated using numbers and percentages.

## Results

According to Table 2, the maximum number of patients (42.8%) belong to the 31-40 age group, followed by 27.1% belonging to the 21-30 age group, and 21.5% belonging to the 51-60 age group. The mean age of the patients is 34.6 years, with a standard deviation of 4.7 years. 

**Table 2:** Age-wise distribution of the study population (n=70).

Age in years	Frequency	Percentage
21–30 years	19	27.1
31–40 years	30	42.8
41–50 years	6	8.6
51–60 years	15	21.5
Total	70	100.0
Mean ± SD (years)	34.6 ± 4.7	

According to Table 3, unilateral abdominal masses (75.71%) were far more prevalent among study participants than bilateral masses (24.28%), constituting only a quarter of the total participants.

**Table 3:** Distribution according to laterality involvement on MRI (n=70).

Laterality/side involved	Number	Percentage
Unilateral	53	75.71
Bilateral	17	24.28
Total	70	100.0

**Figure 1:**
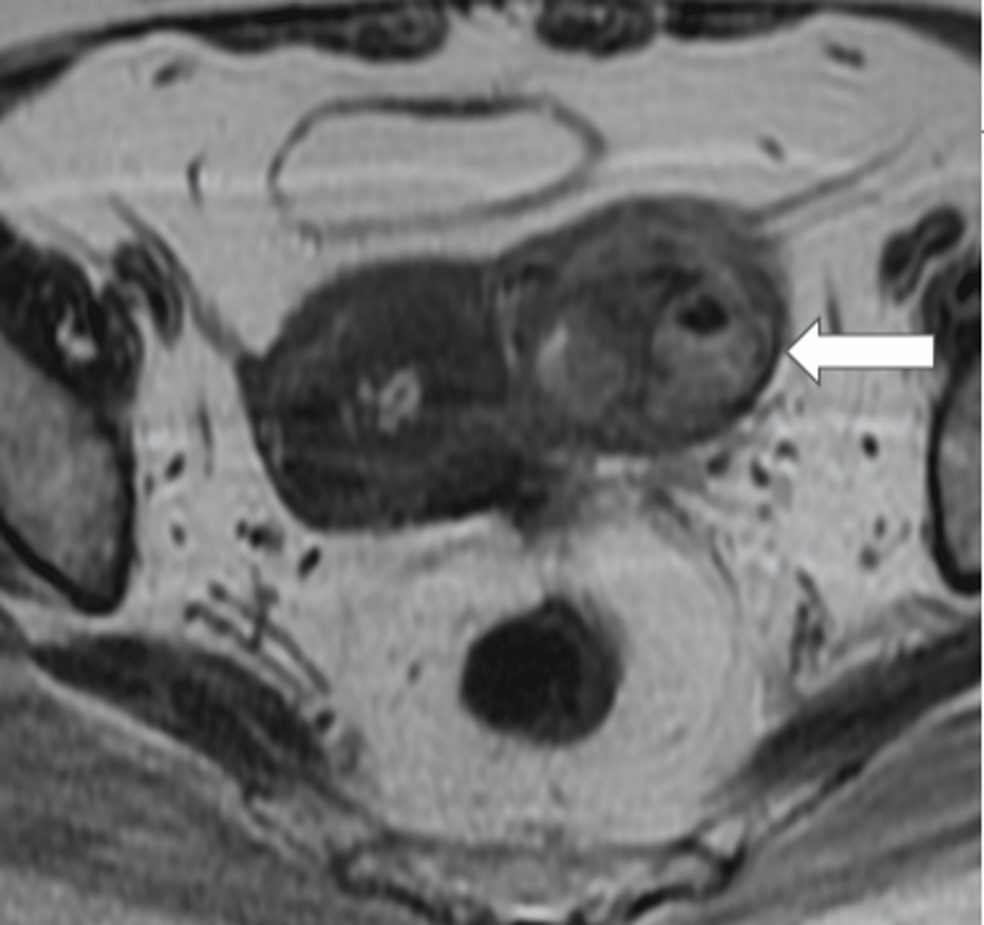
MRI coronal T2-weighted image showing unilateral adnexal lesion on left side.

Figure 1 shows a well-defined heterogenous lesion noted adjacent to the uterus on the left side (unilateral) on Coronal T2-weighted imaging with hypointense areas within (air foci).

According to Table 4, from the MRI findings of study participants, it was depicted that only 18.57% of the abdominal masses of the patients presented with a lesion with nodularity along the wall.

**Table 4:** Distribution according to wall characteristics (nodularity) of the mass on MRI (n=70).

Presence of nodules along the wall	Number	Percentage
Present	13	18.57
Absent	57	18.42
Total	70	100.0

**Figure 2:**
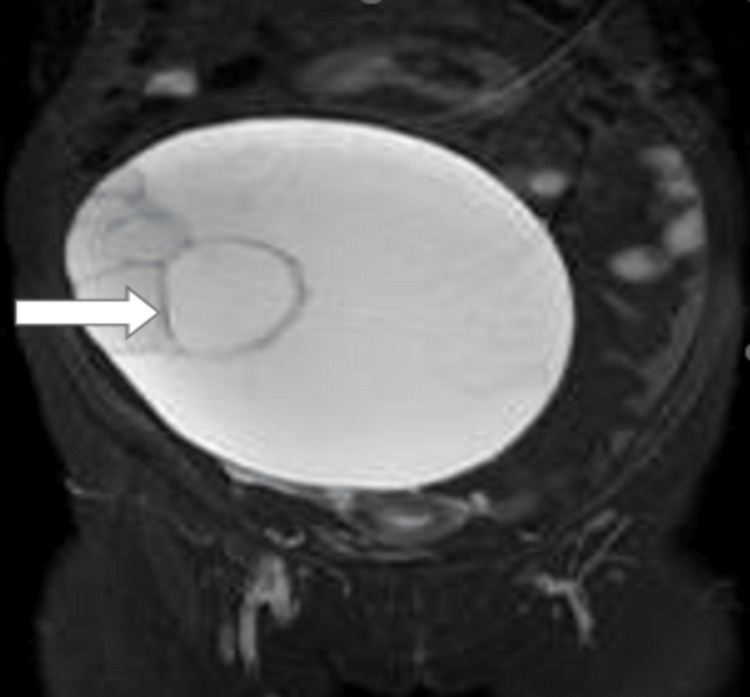
MRI coronal STIR image showing a well-defined hyperintense cystic lesion in adnexa with multiple septations (arrow). STIR: short Tau inversion recovery sequence.

Figure 2 shows a well-defined, hyperintense STIR cystic lesion noted in the adnexa with multiple internal septations within.

According to Table 5, MRI findings suggest that solid and solid cystic masses have an almost similar proportion of approximately 38%. While cystic masses were only 24.28%. The thickness of the septum on MRI when present was 3.0 mm, with a standard deviation of 0.21 mm.

**Table 5:** Distribution according to nature of mass on MRI (n=70).

Nature of mass	Number	Percentage
Solid	27	38.57
Cystic	17	24.28
Solid-cystic	26	37.14
Total	70	100.0

According to Table 6, MRI findings of the abdominal masses suggest that there was enhancement of the lesion in 14.28% of cases, associated ascites in 17.14% of cases, omental deposits in 27.14% of cases, and para-aortic and para-caval lymph node involvement in 28.57% of the cases, indicating severity of the disease.

**Table 6:** Distribution according to other characteristics of mass on MRI (n=70).

Characteristics	Number	Percentage
Enhancement		
Present	10	14.28
Absent	60	85.71
Ascites		
Present	12	17.14
Absent	58	82.85
Omental deposits		
Present	19	27.14
Absent	51	72.85
Lymph nodes		
Present	20	28.57
Absent	50	71.42

**Figure 3:**
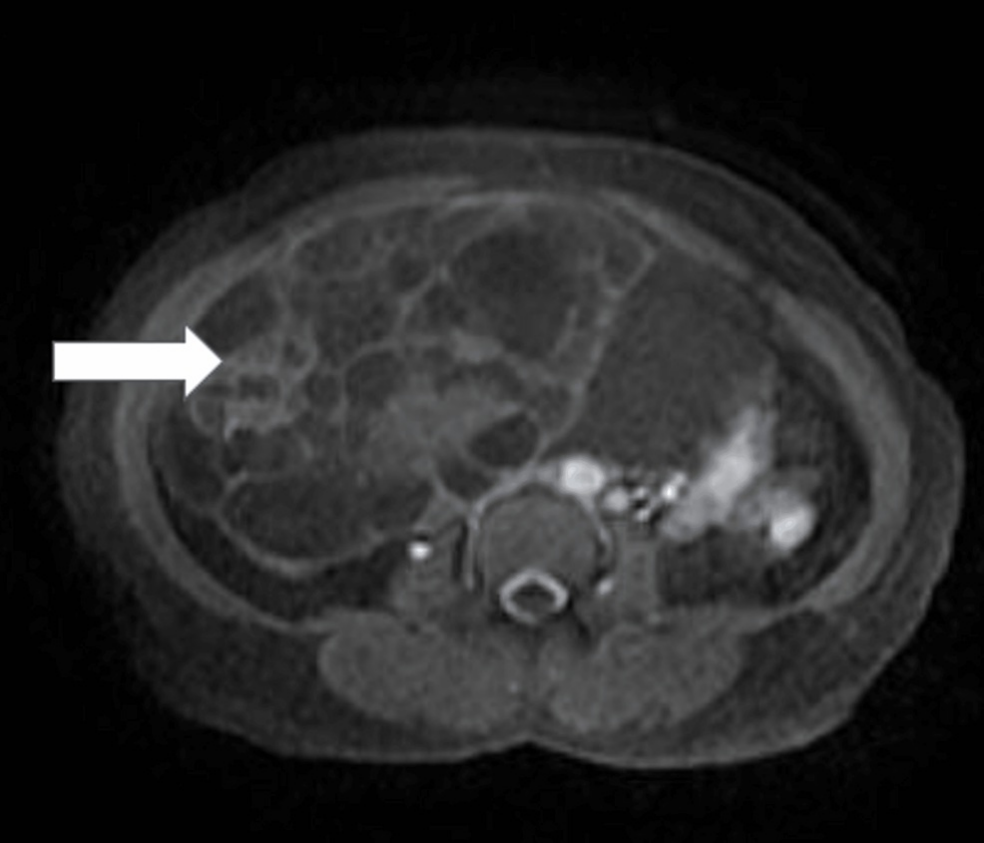
MRI axial post-contrast image showing an adnexal lesion with septal nodular enhancement (arrow).

Figure 3 shows a post-contrast T1-weighted image of an adnexal lesion with septal and nodular enhancement suggestive of a malignant lesion.

According to Table 7, almost half (47.14%) of the patients with abdominal masses included in the study had benign lesions as a MRI finding, followed by non-neoplastic masses (27.14%) and malignant masses (25.71%).

**Table 7:** Distribution according to type of lesion on MRI (n=70).

Type of lesion on MRI	Number	Percentage
Non-neoplastic	19	27.14
Benign	33	47.14
Malignant	18	25.71
Total	70	100.0

According to Table 8, during the histopathology examination (HPE) examination of the abdominal masses, it was observed that there were non-neoplastic as well as neoplastic lesions. Non-neoplastic lesions included para-ovarian cysts, which constituted 10% of the abdominal masses, followed by Follicular cysts (8.57%), endometrioma, and tubo-ovarian abscesses, constituting 4.28% each. Among the surface epithelial tumors, benign serous cystadenomas constituted a major portion of abdominal masses, i.e., 11% of all the abdominal masses diagnosed. Other masses of surface epithelial origin include mucinous cystadenocarcinoma (11.42%), papillary serous cystadenocarcinoma (8.57%), a few low-grade mucinous cystadenocarcinomas, surface epithelial carcinoma of the ovary, and benign mucinous cystadenoma. Germ cell tumors included ovarian dysgerminoma (8.57%), followed by immature and mature germ cell tumors. Sex cord-stromal tumors diagnosed were fibroma (5.71%) and fibrothecoma (7.14%).

**Table 8:** Distribution of lesions according to the type of lesion on histopathology examination (n=70).

Type of lesion on HPE	Frequency	Percentage
Non-neoplastic lesions		
Para ovarian cyst	7	10
Follicular cyst	6	8.57
Endometrioma	3	4.28
Tubo-ovarian abscess	3	4.28
Neoplastic lesions		
Surface epithelial-stromal tumors		
Benign serous cystadenoma	11	15.71
Borderline serous cystadenoma	1	1.42
Benign mucinous cystadenoma	1	1.42
Surface epithelial carcinoma ovary	2	2.85
Papillary serous cystadenocarcinoma	6	8.57
Low grade mucinous cystadenocarcinoma	2	2.85
Mucinous cystadenocarcinoma	8	11.42
Germ cell tumors		
Immature germ cell tumors	3	4.28
Mature germ cell tumors	2	2.85
Ovarian dysgerminoma	6	8.57
Sex cord stromal tumors		
Fibroma	4	5.71
Fibrothecoma	5	7.14
Total	70	100.0

Table 9 depicts that all the lesions characterized as malignant on MRI were diagnosed as malignant on HPE. Therefore, in comparison with HPE findings, it was observed that there was a statistically significant association (p=0.00) between the two diagnostic modalities. This shows that the MRI findings were well correlated with the HPE findings.

**Table 9:** Cross tabulation of histopathology examination and MRI findings (n=70).

MRI	HPE			
	Malignant	Benign	Total	
Malignant	18	0	18	*p value = 0.00 (Fishers’ exact test)
Benign and non-neoplastic lesions	6	46	52	
Total	24	46	70	

According to Table 10, it was observed that the sensitivity for MRI was 75% and the specificity was 100%. The positive predictive value was found to be 100%, whereas the negative predictive value was 88.46%. The overall diagnostic accuracy of MRI was 91.43%.

**Table 10:** Diagnostic accuracy of MRI as compared to histopathology examination.

Variables	Percentage
Sensitivity	75.0%
Specificity	100.0%
Positive predictive value	100.0%
Negative predictive value	88.46%
Overall diagnostic accuracy	91.43%

## Discussion

In the present study, out of 70 recruited females, approximately 70% belonged to the 21-40 age group, i.e., the active reproductive age group, and 21.5% belonged to the 51-60 age group. The mean age of females presenting with adnexal masses in the present study was 34.6 ± 4.7. The reproductive age group constitutes more hormonal fluctuations and stress related to work and family responsibilities, which may be contributing factors to the adnexal mass incidence [8-11]. Different types of adnexal masses diagnosed in the present study were non-neoplastic masses such as para-ovarian cysts (10%), follicular cysts (8.57%), endometrioma (4.28%), tubo-ovarian abscess (4.28%), surface epithelial tumors, germ cell tumors, and sex cord-stromal tumors.

Among the surface epithelial tumors, benign serous cystadenomas constituted the major portion of abdominal masses, i.e., 11% of all the abdominal masses diagnosed. Other masses of surface epithelial origin include mucinous cystadenocarcinoma (11.42%), papillary serous cystadenocarcinoma (8.57%), a few simple serous cystadenomas, low-grade mucinous cystadenocarcinomas, surface epithelial carcinoma of the ovary, and benign mucinous cystadenoma. Germ cell tumors included ovarian dysgerminoma (8.57%), followed by immature and mature germ cell tumors. Sex cord-stromal tumors diagnosed were fibroma (5.71%) and fibro-thecoma (7.14%). In a study, Debbama et al. found various benign adnexal masses such as dermoid, endometrioma, serous cystadenocarcinoma, Brenner tumor, hydrosalpinx, simple cyst, para-ovarian cyst, fibroma, endometriosis, and malignant masses like papillary cystadenocarcinoma, dysgerminoma, metastasis, granulosa cell tumor, adenocarcinoma, clear cell carcinoma, and immature teratoma [12].

The present study observed that more than 70% of adnexal masses were unilateral. In advanced cancers, tumor laterality can be considered an independent prognostic factor. Due to peritoneal cavity flow pattern dynamics or differential flow patterns of the right and left ovarian veins, patients with left-sided ovarian cancer have a better prognosis than those with right-sided ovarian cancer, according to a few studies [13]. Bilateral cancers show more invasiveness to the surrounding structures than unilateral masses. Also, due to limited invasion in unilateral lesions, the rate of residual tumors or missed lesions is lower. The bilaterality of the masses itself suggests synchronous tumorigenesis and metastasis from one side to another. A higher number of unilateral masses in the present study indicates less invasiveness of the lesions [14].

As per the MRI findings of the study, only 18.57% of the masses presented with specific wall findings or nodularities. The nature of masses on MRI was 38.57% solid, 24.28% cystic, and 37.14% solid cystic. MRI also depicted characteristics of masses, such as enhancement in 15% of masses, ascites in 17% of masses, omental deposits in 27% of the masses, and the presence of lymph nodes in 29% of the masses. The origin of mass, invasiveness, septations, and nodularity can be characteristically better seen on MRI, as it includes sections at various levels and aspects. Different intensities for fat, hemorrhage, and fluid give characteristically better diagnoses than ultrasonography. However, according to the study by Sohaib et al., vegetation or nodules in a cystic lesion, the presence of ascites, a maximal diameter higher than 6 cm, and necrosis in a solid lesion are the strongest predictive indicators of malignancy in MR imaging [15].

According to the present study, MRI reveals 27% non-neoplastic, 47% benign, and 26% malignant lesions, whereas ultrasonography (USG) indicates 27% non-neoplastic, 49% benign, and 24% malignant lesions. Histopathology, the gold standard for identifying and classifying pathological masses, positively identified lesions and classified them as non-neoplastic lesions, surface epithelial-stromal tumors, germ cell tumors, and sex cord-stromal tumors. In the present study of 70 cases with adnexal masses, there was a strong positive correlation between MRI and HPE findings.

In a study by Jayanthan et al., MRI was found to be a far superior imaging modality when it comes to determining the origin of adnexal lesions. The authors of the study were able to correctly identify the origin of the lesions in each case, despite the unfavorable characteristics of the masses and the surrounding environment. The results of the study further demonstrate the importance of using MRI for the diagnosis and treatment of adnexal lesions, as it can provide more accurate and reliable results than other imaging modalities [16]. T1- and T2-weighted imaging is critical in MRI for precise tissue characterization. On T1-weighted imaging with and without fat suppression, lipids and blood are easily identified and distinguished. Fat must be demonstrated using both conventional and fat-suppressed T1-weighted imaging, since the latter aids in distinguishing fat from blood products as the source of the high T1 signal intensity. T2-weighted imaging aids in identifying endometriomas with very low signal intensity, indicating blood breakdown products from repetitive cyclical bleeding, or fibrous tissue with extremely low signal intensity in an ovarian fibrous tumor (i.e., Brenner tumor, ovarian fibroma, or fibrothecoma) [17].

Advanced imaging is required for a better understanding of adnexal masses, such as ovarian cysts greater than 7 cm. While serous cystadenomas typically exhibit MRI signals indicative of clear fluid, mucinous cystadenomas typically manifest as multilocular lesions with thin, regular walls and septae. By separating the healthy ovary from the cyst and seeing them side by side, it is also simple to distinguish between para-ovarian cysts. MRI is more accurate than ultrasound at locating disease locations in endometriosis cases [16,18].

The main limitation of our study was the small sample size due to COVID pandemic constraints, as a larger sample size will help in generalizing the study findings to the population.

## Conclusions

MRI provides the added advantage of visualization of the tumor matrix with differential identification of the fatty and cystic tissue through heterogeneous signals and enhancement indicating aggressiveness and local spread.

MRI has greater diagnostic accuracy when compared to USG, with HPE as the gold standard for discriminating between benign and malignant adnexal masses. MRI has the additional advantage of visualizing the tumor matrix, allowing for differentiated detection of fatty and cystic tissue. MRI also facilitates the differentiation of malignancies by providing information on the margins of the tissue and the microvascular invasion, as well as by providing heterogeneous signals and enhancement that indicate the aggressiveness and local spread of the disease.
